# A Simulated Shift Work Schedule Does Not Increase DNA Double-Strand Break Repair by NHEJ in the *Drosophila* Rr3 System

**DOI:** 10.3390/genes13010150

**Published:** 2022-01-15

**Authors:** Lydia Bergerson, Caleb Fitzmaurice, Tyler Knudtson, Halle McCormick, Alder M. Yu

**Affiliations:** Department of Biology, University of Wisconsin-La Crosse, La Crosse, WI 54601, USA; bergerson5651@uwlax.edu (L.B.); fitzmaur.cale@uwlax.edu (C.F.); jeschke.tyler@uwlax.edu (T.K.); mccormick6635@uwlax.edu (H.M.)

**Keywords:** circadian rhythm, DNA repair, mutation, NHEJ, *Drosophila*

## Abstract

Long-term shift work is widely believed to increase the risk of certain cancers, but conflicting findings between studies render this association unclear. Evidence of interplay between the circadian clock, cell cycle regulation, and DNA damage detection machinery suggests the possibility that circadian rhythm disruption consequent to shift work could alter the DNA double-strand break (DSB) repair pathway usage to favor mutagenic non-homologous end-joining (NHEJ) repair. To test this hypothesis, we compared relative usage of NHEJ and single-strand annealing (SSA) repair of a complementary ended chromosomal double-stranded break using the Repair Reporter 3 (Rr3) system in *Drosophila* between flies reared on 12:12 and 8:8 (simulated shift work) light:dark schedules. Actimetric analysis showed that the 8:8 light:dark schedule effectively disrupted the rhythms in locomotor output. Inaccurate NHEJ repair was not a frequent outcome in this system overall, and no significant difference was seen in the usage of NHEJ or SSA repair between the control and simulated shift work schedules. We conclude that this circadian disruption regimen does not alter the usage of mutagenic NHEJ DSB repair in the *Drosophila* male pre-meiotic germline, in the context of the Rr3 system.

## 1. Introduction

Engagement in shift work that involves light during subjective night has been identified as a probable carcinogen [1]. Long-term shift workers have been suggested to be at heightened risk for cancers of the breast [2] (reviewed in [3,4]), prostate [5], and colon [6]. However, a recent combined prospective study and meta-analysis failed to support an association between shift work and breast cancer [7], and another recent study failed to support a link between night-shift work and prostate cancer [8]. Thus, the question of whether night-shift work is indeed a risk factor for cancer in humans remains unclear.

The most immediate effect of night-shift work is the disruption of the circadian rhythm by exposure to light during subjective night, inconsistent sleep schedules, and irregular mealtimes (reviewed in [9,10,11]). Potential direct mechanistic links have been identified between circadian rhythm disruption and tumor promotion, including increased expression of oncogenes [12], altered levels of inflammation-associated proteins [13,14], and disruption of the cell cycle (reviewed in [15]). Conversely, the expression of circadian clock genes has been noted to be disrupted in tumor cells (reviewed in [16]). A prominent indirect mechanistic link between long-term shift work and tumor promotion is increased obesity [17,18,19,20] and metabolic syndrome [17,21,22]. At the molecular level, circadian rhythm disruption produces metabolic abnormalities in rats [23], and impairs insulin sensitivity in human subjects [24]. Behavioral risk factor profiles associated with shift workers have been proposed to play a role in this [10], including increased smoking [25] and alcohol consumption [26] among shift workers.

The possibility of a mechanistic link between circadian rhythm disruption and tumor initiation is less well examined. The key event in tumor initiation is thought to be the accumulation of mutations via unrepaired or misrepaired DNA damage (reviewed in [27]). Some evidence does suggest that circadian rhythm disruption may impair DNA damage repair. Night-shift workers show decreased levels of 8-hydroxydeoxyguanosine excretion in the urine, which could be explained by the reduced ability to remove this oxidative lesion from the genome [28]. Another study showed that PER1 (Period Circadian Regulator 1), a key component of the core circadian clock, physically interacts with the ataxia telangiectasia-mutated (ATM) protein, which is crucial for detecting and initiating the cellular response to DNA double-strand breaks (DSBs). The same study showed that ATM also interacts with CHEK2 (checkpoint kinase 2), which mediates the cell cycle response to DNA damage. Additionally, PER1 sensitizes cells to ionizing radiation-induced apoptosis, suggesting a directly protective role of circadian clock proteins against carcinogenic transformation [29]. Underscoring the possibility of a role for the circadian clock in the cellular response to DNA DSBs, a different core clock protein, TIM (timeless circadian regulator), was shown to interact with the DNA damage-sensing proteins ATR (ATR serine/threonine kinase) and ATRIP (ATR-interacting protein), and with the cell cycle regulator CHEK1(checkpoint kinase 1). Furthermore, the interactions between TIM, ATRIP, and CHEK1 were enhanced by hydroxyurea-induced DNA damage [30].

A connection between the circadian clock and the cellular response to DSBs is especially salient because DSBs are a particularly genotoxic lesion. Since both strands are compromised, the potential for mutation is high. Genetic disorders that impair the ability to detect or repair DSBs are typically cancer prone syndromes (reviewed in [31]). DSBs can be repaired by a number of different pathways with varying degrees of fidelity (reviewed in [32]). The outcome of homologous recombination repair (HRR) depends on choice of template. HRR templated from an intact sister chromatid during late S or G2 can restore the original sequence, whereas HRR from the homolog in a heterozygous individual will result in the loss of heterozygosity (gene conversion). DSBs flanked by repeated sequences may be repaired by annealing of the complementary sequences with loss of the intervening region, in a process called single-strand annealing (SSA) (reviewed in [33]). Non-homologous end-joining (NHEJ) DSB repair rejoins the ends directly, without consulting homologous sequence external to the break. In the case of a simple break with chemically undamaged DNA ends, the outcome of NHEJ may be a reconstitution of the original sequence. However, sequence changes at the repair site are common (reviewed in [34]).

Repair pathway choice depends on a number of factors, including capacity to detect the break and cell cycle phase [35]. Evidence that the circadian clock machinery interacts with key proteins in both of these processes suggests the possibility that circadian rhythm disruption could influence DSB repair pathway choice, potentially leading to an increase in mutagenic repair. This would constitute a mechanistic link between circadian rhythm disruption and tumor initiation.

To test the hypothesis that circadian rhythm disruption can alter DNA DSB repair pathway choice, we used the Repair Reporter 3 (Rr3) system [36] to examine the relative use of SSA and NHEJ repair of a complementary ended chromosomal DSB in fruit flies kept on a conventional 12:12 light:dark (L:D) schedule, and flies kept on an 8:8 L:D schedule. The Rr3 system allows rapid visual identification of the DSB repair mechanism usage via expression of the fluorescent DsRed protein. The Rr3 construct contains a DsRed gene that is interrupted by a 147 bp repeat flanking a cut site for the I-*Sce*I endonuclease. Repair of the endonuclease-induced break by SSA activates DsRed expression, and repair by NHEJ does not. Therefore, the repair mechanism can be identified by a simple visual screen. Flies containing both Rr3 and an I-*Sce*I expression construct are generated by appropriate crosses. Breakage and repair events take place in the male pre-meiotic germline, and are recovered by appropriate crosses to be scored in the next generation.

We found that the 8:8 L:D schedule effectively abrogated normal activity rhythms. No difference was seen in the relative use of SSA and NHEJ between flies kept on the 12:12 and 8:8 schedules. We conclude that in the Rr3 system, the choice of NHEJ versus SSA repair is not affected by this method of circadian rhythm disruption.

## 2. Materials and Methods

*Fly stocks and husbandry. w^1118^* flies were the kind gift of F. Rob Jackson (Tufts University). Rr3 and P{UIE} flies [36] were the kind gift of Osamu Suyari (University of Oxford). Flies were fed Bloomington formula cornmeal-agar medium (Genessee Scientific, San Diego, CA) prepared according to the manufacturer’s instructions with propionic acid. Flies were maintained on 12:12 or 8:8 light:dark (L:D) schedules at 25 °C in programmable incubators (Percival Scientific, Perry, IA) and handled under light carbon dioxide or ether anesthesia. Full genotypes of all flies are provided in Table 1.

*Activity monitoring.* Time courses of locomotor activity were recorded using the Drosophila Activity Monitor (DAM) system (Trikinetics, Waltham MA) [37]. DAM5M monitors were used in all experiments. Recently eclosed male flies were individually loaded into food-containing activity tubes under light anesthesia and allowed to recover for at least 24 h before recording data for analysis. Activity was recorded as total beam crossings per five-minute bin, using Trikinetics DAM System 3 software. Data files were validated using DAM File Scan software and exported as CSV files. Data were analyzed with the ShinyR-DAM v3.1 software package [38], using the publicly available web interface at https://karolcichewicz.shinyapps.io/shinyr-dam/ (accessed on 8 January 2022). Circadian period length was analyzed by the Chi-square periodogram [39] implementation of ShinyR-DAM. Significance threshold indicated by the diagonal line in periodograms should be interpreted with caution, as fewer than 10 days of data were analyzed in each case.

*RT-qPCR*. P{UIE} flies (2–3 per sample) were collected under light ether anesthesia, immediately ground in Trizol LS reagent (Thermo Fisher Scientific, Waltham, MA, USA) and stored at −80°. RNA was extracted with chloroform and further purified with the RNA Clean and Concentrator–5 kit (Zymo Research, Irvine, CA, USA) with on-column DNase I digestion carried out according to the manufacturer’s instructions. 100 ng of RNA was used for reverse transcription with the iScript Advanced cDNA kit (BioRad, Hercules, CA, USA). qPCR was carried out according to standard methods in a BioRad CFX96 Touch 1000 thermocycler using SsoAdvanced Universal SYBR Green Supermix (Bio-Rad), according to the manufacturer’s instructions with 0.5 µL of cDNA per reaction. Primers used to amplify the *period* gene were Per-F (5′–CAGCTGCAGCAACAGCCAGTCG–3′) and Per-R (5′–GGCCTGCGTCGAGGGCTTGC–3′). The ribosomal gene RpL32 was used as a constant expression control, with the primers RpL32-F (5′–GCCCAAGATCGTGAAGAAGC–3′) and RpL32-R (5′–CGACGCACTCTGTTGTCG–3′). Relative expression was calculated using the double delta Cq method [40], using the average Cq of t = 0 samples as the untreated reference.

*The Rr3 construct.* The Rr3 DSB repair reporter construct [36] consists of a DsRed gene that has been rendered nonfunctional by insertion of the 13 bp recognition site for the I-*Sce*I endonuclease, flanked by a 147 bp repeat. The Rr3 construct additionally contains a functional copy of the *white* gene, which allows the presence of the Rr3 construct to be identified in the *white* mutant background via examination of eye color. When the Rr3 construct is cut by I-*Sce*I, the resulting complementary ended DSB can be repaired by SSA, NHEJ, or HRR. SSA repair using the 147 bp repeat restores DsRed gene expression. Accurate NHEJ restores the original sequence, which can be cut again. Inaccurate NHEJ that alters the sequence of the I-*Sce*I site prevents further DSB induction without restoring DsRed expression. HRR from an intact sister chromatid reconstitutes the original sequence, which can be cut again, and HRR from the homolog results in gene conversion and complete loss of the Rr3 site. All experiments used an Rr3 construct integrated into chromosome 2 at 48C. Full details of the construction of Rr3 are given in [36].

**Figure 1:**
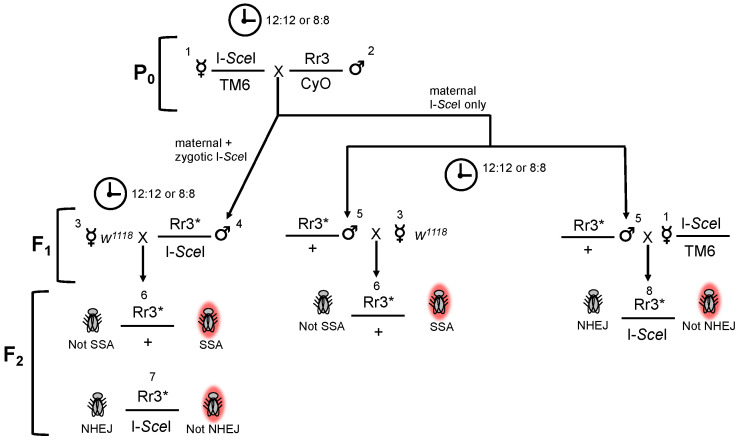
Multi-generational cross scheme used to assess the effect of non-24 h L:D schedule on relative use of SSA and NHEJ repair in the male pre-meiotic germline. Asterisk indicates an Rr3 construct that has been exposed to I-*Sce*I endonuclease. Full genotypes of numbered flies are indicated in Table 1 below.

*Induction of double-strand breaks and quantitation of repair events.* When male flies harboring an Rr3 construct are crossed to female flies expressing the I-*Sce*I endonuclease under a constitutive ubiquitin promoter (Figure 1, P_0_ cross), F_1_ progeny undergo repeated cycles of DSB induction and repair in all cells. The timing of DSB induction depends on whether endonuclease is provided by maternal effect only, or by both maternal effect and zygotic expression of I-*Sce*I. The chromosome containing the I-*Sce*I expression construct contains a dominant mutant allele of the gene Stubble (*Sb*; Table 1), allowing flies harboring the I-*Sce*I expression construct to be identified via examination of bristle length. In F_1_ progeny that inherit both the Rr3 and an I-*Sce*I gene, DSB induction and repair cycles begin at the commencement of embryonic development, due to maternal effect nuclease, and continue until a repair event mutates or deletes the I-*Sce*I site (Figure 1, F_1_ generation, genotype 4). In F_1_ progeny that inherit only the Rr3, DSB induction is due solely to maternal effect endonuclease in the egg, and continues until an inaccurate repair event takes place or maternal effect protein is exhausted (Figure 1, F_1_ generation, genotype 5). Relative usage of different DSB repair mechanisms in the F_1_ generation male pre-meiotic germline is quantitated by recovering individual repair events via appropriate crosses, and scoring F_2_ individuals for DsRed expression.

For F_1_ germlines that have been exposed to both maternal effect and zygotically expressed endonuclease (Figure 1, genotype 4), germline repair events are recovered and counted via crossing to virgin *w^1118^* females. Germline SSA events are quantitated as the percentage of progeny that express DsRed out of those that did not inherit an I-*Sce*I gene (Figure 1, genotype 6). DsRed-expressing F_2_ flies that inherited both Rr3 and an I-*Sce*I gene (Figure 1, genotype 7) are not considered when calculating SSA, because they may express DsRed due either to germline SSA or somatic SSA during the development of a fly that inherited an intact Rr3 construct. Germline NHEJ is quantitated as the percentage of non DsRed-expressing F_2_ progeny out of those that inherited both an Rr3 and an I-*Sce*I gene (Figure 1, genotype 7). Flies that inherited both will express DsRed, either from a germline SSA event or somatic SSA of an inherited intact construct, unless a germline inaccurate NHEJ event has prevented further DSB induction.

For F_1_ germlines that have been exposed to maternal effect endonuclease only (Figure 1, genotype 5), the logic underlying scoring repair events is the same, but SSA and NHEJ are scored using separate crosses to avoid exposing F_1_ events that resulted in accurate repair to an additional round of endonuclease-induced cutting during F_2_ embryonic development when scoring SSA repair. SSA events are recovered and identified in the F_2_ generation via crosses to *w^1118^* females, as described above (Figure 1, genotype 6). NHEJ events are recovered in the F_2_ generation via crossing to I-*Sce*I-expressing females, and scored as described above (Figure 1, genotype 8).

F_2_ offspring were scored for DsRed expression using a Nikon SMZ 1500 fluorescence microscope with a DsRed filter. Vials that produced fewer than 20 F_2_ progeny were excluded from analysis. Data were graphed and statistical analyses carried out in Excel and Prism software.

## 3. Results

### 3.1. A Non-24 H Light:Dark Schedule Disrupts Activity Rhythms

**Figure 2:**
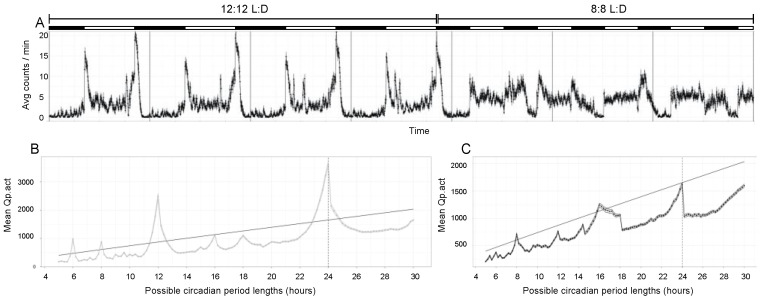
Actimetric analysis of male *w^1118^* flies (n = 32) under 12:12 L:D and 8:8 L:D conditions. Error bars in all panels indicate standard error. (**A**) Flies show the expected rhythmic bimodal activity pattern under 12:12 L:D, which is degraded when the lighting schedule is shifted to 8:8 L:D. White and black bars above panel indicate periods of light and darkness. Vertical bars in panel indicate 24 h elapsed time. Activity was averaged in five-minute bins. (**B**) Chi-square periodogram for 12:12 L:D shows a clear major probability peak at 24 h, consistent with a 24 h period in activity rhythms. (**C**) Chi-square periodogram for 8:8 L:D shows no clear peak, consistent with reduced rhythmicity. The diagonal line in panels (**B**,**C**) indicating statistical significance cutoff should be interpreted with caution, as fewer than 10 days of data were analyzed. Activity data file is available as Table S2.

Under 12:12 L:D conditions, flies typically show 24-h rhythmicity in locomotor activity, with bimodal daily peaks at lights on and lights off (reviewed in [41]). To ensure that the simulated shift work schedule (8:8 L:D) disrupted the daily rhythms, we used the Trikinetics Drosophila Activity Monitor (DAM) system to compare locomotor rhythms of flies under 12:12 and 8:8 L:D (Figure 2). Under 12:12 L:D conditions, *w^1118^* flies showed the expected bimodal activity peaks (Figure 2A). Chi-square periodogram analysis [39] of activity data from the 12:12 L:D days showed a major peak at 24 h, consistent with expected rhythmicity under 12:12 L:D conditions. (Figure 2B). When the lighting schedule was shifted to 8:8 L:D, the bimodal peak pattern was degraded (Figure 2A) and the periodogram showed no clear single peak (Figure 2C), consistent with a substantial reduction in rhythmicity under 8:8 L:D conditions. Similar results were obtained with p{UIE} flies (Figure S1).

To assess the function of the circadian clock on the molecular level under 12:12 and 8:8 L:D conditions, we carried out an RT-qPCR expression time course of the *period* gene, a key component of the negative feedback limb of the *Drosophila* circadian clock [42]. Results are shown in Figure S2. Under 12:12 conditions, *period* expression in P{UIE} flies showed the expected daily oscillations, albiet with a 4 h phase shift from the expected expression peak and nadir. In contrast, *period* expression in flies kept under 8:8 conditions did not show clear rhythmicity, and exhibited substantial inter-sample variability, suggesting that the 8:8 L:D schedule does disrupt the circadian clock at the molecular level.

### 3.2. 8:8 L:D Does Not Alter Relative Usage of NHEJ and SSA When Endonuclease Is Supplied by Maternal Effect Only

We wished to examine the effect of long-term circadian rhythm disruption on DNA double-strand break repair. However, in the Rr3 system with endonuclease expression controlled by a constitutive promoter, a substantial proportion of repair events occur early in the life cycle, before the effects of sustained circadian rhythm disruption can accumulate. To circumvent this technical limitation, we examined repair events mediated by maternal effect endonuclease in embryos produced by female flies that had been raised under either 12:12 or 8:8 conditions. We reasoned that before the onset of zygotic transcription, the repair capacity of the embryo would reflect that of the female parent.

**Figure 3:**
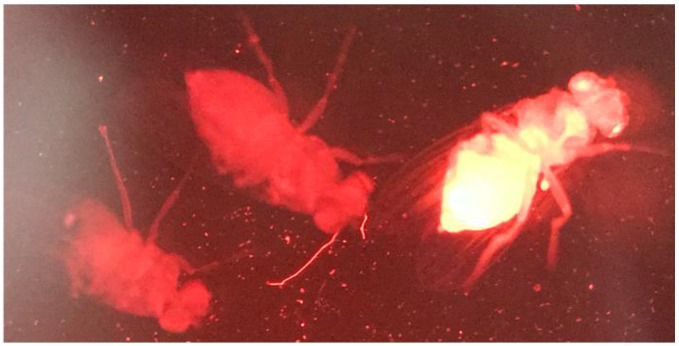
Example of DsRed-expressing (right) and -non-expressing (left) flies. The photograph was cropped to remove the microscope objective edges visible at the border of the image.

**Figure 4:**
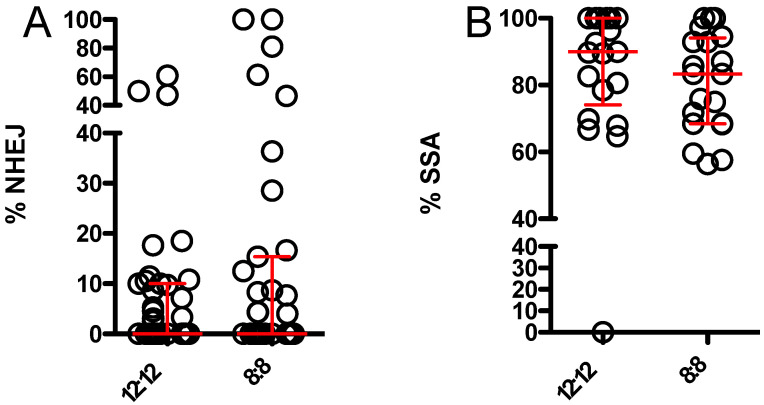
Relative usage of NHEJ and SSA DSB repair pathways with maternal effect endonuclease only in flies maintained under 12:12 versus 8:8 L:D. Each symbol represents the offspring of one independent male germline. Red bars and whiskers represent median and interquartile range. Medians were compared via Mann–Whitney test. (**A**) Relative use of inaccurate NHEJ showed no difference between flies maintained under 12:12 L:D (n = 39) and 8:8 L:D (n = 35) (*p* > 0.75). (**B**) Relative use of SSA showed no difference between flies maintained under 12:12 L:D (n = 21) and flies maintained under 8:8 L:D (n = 20) (*p* > 0.58). Data used to construct this figure are available in Table S4.

DsRed-expressing and -non-expressing flies were readily distinguished (Figure 3). Results for percent NHEJ and SSA repair after maternal effect DSB induction are shown in Figure 4. NHEJ repair was relatively uncommon under both conditions, with a small number of germlines under both conditions showing “jackpot effects”, due to an NHEJ event early in embryogenesis. No significant difference in NHEJ repair was seen. Use of SSA repair showed a modest decrease under 8:8 conditions, but the difference did not reach statistical significance.

### 3.3. 8:8 L:D Does Not Alter Relative Usage of NHEJ and SSA When Endonuclease Is Supplied by Maternal Effect and Zygotic Expression

**Figure 5:**
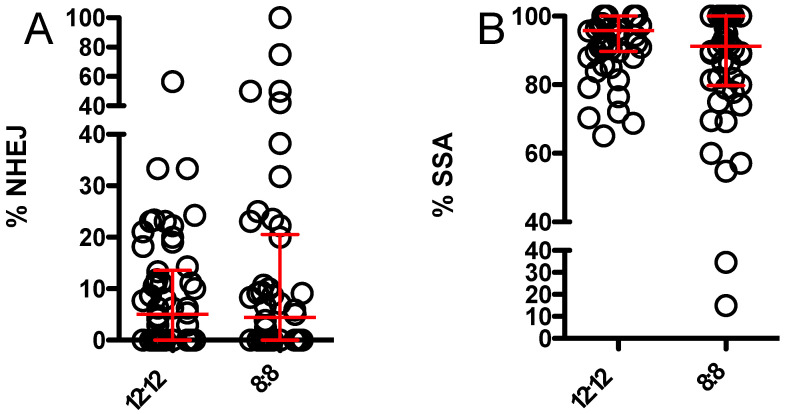
Relative usage of NHEJ and SSA DSB repair pathways with maternal effect and zygotic endonuclease in flies maintained under 12:12 versus 8:8 L:D. Each symbol represents the offspring of one independent male germline. Red bars and whiskers represent median and interquartile range. Medians were compared via Mann–Whitney test. (**A**) Relative use of inaccurate NHEJ showed no difference between flies maintained under 12:12 L:D (n = 54) and 8:8 L:D (n = 46) (*p* > 0.91). (**B**) Relative use of SSA showed no difference between flies maintained under 12:12 L:D (n = 54) and flies maintained under 8:8 L:D (n = 46) (*p* > 0.13). Data used to construct this figure are available in Table S4.

The *Drosophila* circadian clock begins to function during embryogenesis and can respond to light inputs during development [43]. Therefore, we reasoned that non-24 h light:dark cycles during development could, in principle, have an effect on DSB repair during this time. To examine this, we quantitated the relative use of SSA and NHEJ in flies exposed to a combination of maternal effect and zygotically expressed endonuclease. The results are shown in Figure 5. A modest decrease in use of SSA repair was seen in 8:8 relative to 12:12 L:D flies, but this difference did not reach statistical significance.

## 4. Discussion

Human studies have produced conflicting results regarding whether long-term shift work should be considered a carcinogen. Resolution of this uncertainty will require elucidation of any mechanistic links between long-term shift work and initiation or promotion of tumorigenesis. Although potential links between circadian rhythm disruption and tumor promotion have been investigated in some detail, little previous work has directly examined the possibility of a link between circadian rhythm disruption and tumor initiation via increased usage of mutagenic DNA double-strand break repair.

Previously published studies support the notion that susceptibility to DNA damage is time-of-day-dependent, and this dependence requires a functional circadian clock. For example, Plikus et al. [44] showed in mice that the genotoxic effect of ionizing radiation on hair matrix cells varies with time of day, and that this effect is abolished in mice homozygous for a loss of function mutation in a core circadian clock gene. This effect was further linked to an interplay between the circadian clock and the G2/M checkpoint. Conversely, it has been shown that loss of function mutations in circadian clock components can enhance hyperproliferative phenotypes, pointing to a dysregulation of cell cycle control (reviewed in [45]). Since DSB repair pathway choice depends heavily on the cell cycle phase [32], it therefore stands to reason that cell cycle phase disruption consequent to circadian rhythm disruption could impact DNA double-strand break repair pathway choice. However, to our knowledge, no previous studies have directly asked whether loss or disruption of circadian rhythms can affect DSB repair pathway choice.

To investigate the possibility that circadian rhythm disruption could affect DNA double-strand break repair pathway choice, we compared relative usage of SSA and NHEJ in repairing a complementary ended DNA double-strand break between flies maintained on a conventional 12:12 light:dark schedule and a schedule designed to simulate rotating 8-hour shifts. Surprisingly, we observed no difference in usage of NHEJ repair between these conditions.

Further investigation will be required to determine whether the results from this study can be generalized to other break and cell types. DSB repair pathway choice is strongly dependent on the sequence surrounding and structure of the break [32]. The location of the break in euchromatin versus heterochromatin also plays a role in repair pathway choice (reviewed in [46]) The Rr3 construct is designed to promote SSA repair, but not all breaks will have the necessary flanking repeats to carry out SSA. Additionally, it is possible that circadian rhythm disruption could increase NHEJ at the expense of a different pathway, such as HRR. Further studies with the Rr3 construct will investigate the effect of circadian rhythm disruption on HRR from the homologous chromosome. Ultimately, to definitively answer the question of whether circadian rhythm disruption impacts DSB repair pathway choice, it will be necessary to examine a variety of breaks in different sequence and chromatin contexts.

One additional consideration for interpreting results in the Rr3 system is a possible effect of the mutant alleles used as visible genetic markers. In particular, the P{UIE} flies are homozygous for a mutant allele of the *ebony* gene, which has been shown to be involved in circadian locomotor output [47]. We do not consider it likely that this will have an effect on DNA repair, as *ebony* mutant flies have been shown to have normal circadian clock function [47]. However, this consideration underscores the need for investigation of the interaction of the circadian clock with DNA damage repair in multiple different experimental systems.

It is also possible that the cells comprising the embryonic or mature male pre-meiotic germline are not vulnerable to the deleterious effects of circadian rhythm disruption. We do note that core circadian clock genes are expressed in the *Drosophila* testis, and mutations in core clock genes impair male fertility [48]. Similarly, the core clock gene *Period* is expressed in the female *Drosophila* ovary [49], although it has been argued to serve a function in developmental, rather than circadian, timekeeping [50]. Alternatively, even if the 8:8 L:D schedule does not disrupt core circadian clock protein function in the germline directly, circadian rhythm disruption could potentiate organism-level redox imbalance (reviewed in [51]), which could dysregulate signaling pathways mediated by ATM (reviewed in [52]). Thus, although this study does not support a role for circadian clock function in influencing DSB repair pathway choice, it also does not rule out the possibility of such a link. Further research will be necessary to extend these findings to other damage types, DSB repair pathways, and break structures.

## Figures and Tables

**Table 1:** Full genotypes of flies described in Figure 1. P{UIE} is an abbreviation for *P{Ubiq::I-SceI}*. Details of the construction of the Rr3 and P{UIE} constructs are given in [36].

	Genotype
1	*w; TM3 Sb P{UIE}72C/TM6 Ubx*
2	*w/Y; al wg^Sp−1^ P{Rr3}48C L sp/CyO*
3	*w^1118^*
4	*w/Y; al wg^Sp−1^ P{Rr3}48C L sp/+; TM3 Sb P{UIE}72C/+*
5	*w/Y; al wg^Sp−1^ P{Rr3}48C L sp/+*
6	*w^1118^/Y; al wg^Sp−1^ P{Rr3}48C L sp/+*
7	*w^1118^/Y; al wg^Sp−1^ P{Rr3}48C L sp/+; TM3 Sb P{UIE}72C/+*
8	*w/Y; al wg^Sp−1^P{Rr3}48C L sp/+; TM3 Sb P{UIE}72C/+*

## Data Availability

Data files used to generate the figures in the main text are available as supplementary information.

## Supplementary Materials

The following supporting information can be downloaded at: https://www.mdpi.com/article/10.3390/genes13010150/s1, Figure S1, Activity data for P{UIE} flies; Figure S2, RT-qPCR of the *period* gene; Table S1: Activity data for Figure 1, Table S2: Activity data for Figure S1; Table S3, RT-qPCR enrichment data; Table S4, Tabulation of repair type percentage by individual.

## Abbreviations

DSB	DNA double-strand break
HRR	homologous recombination repair
NHEJ	non-homologous end joining
Rr3	Repair Reporter 3
SSA	single strand annealing
