# Supplementary material for: A Simulated Shift Work Schedule Does Not Increase DNA Double-Strand Break Repair by NHEJ in the Drosophila Rr3 System
Source: Genes (Basel). 2022 Jan 15;13(1):150. doi: 10.3390/genes13010150 (PMC8774994; doi:10.3390/genes13010150)
Supplement: Supplementary file 1 [file genes-13-00150-s001.zip › Figure S1.pdf]

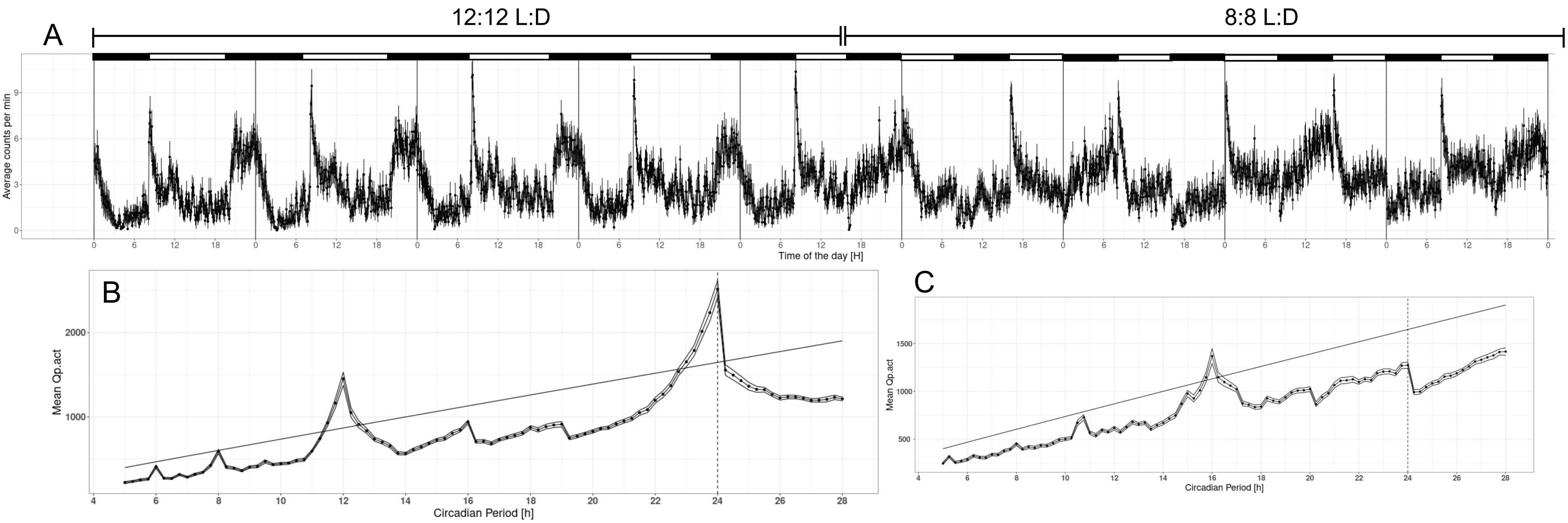

Figure S1. Actimetric analysis of P{UIE} flies (n=32). (a) Flies show the expected bimodal activity peaks under 12:12 L:D conditions, which is degraded when flies are shifted to an 8:8 L:D schedule. (b) Chi-square periodogram of 12:12 L:D activity data shows a peak at a 24 hour period, consistent with normal circadian rhythmicity. (c) Chi-square periodogram of 8:8 L:D activity data shows altered periodicity of locomotor activity rhythms. The data file used to generate these figures is available as Table S1.
