# Supplementary material for: A Simulated Shift Work Schedule Does Not Increase DNA Double-Strand Break Repair by NHEJ in the Drosophila Rr3 System
Source: Genes (Basel). 2022 Jan 15;13(1):150. doi: 10.3390/genes13010150 (PMC8774994; doi:10.3390/genes13010150)
Supplement: Supplementary file 1 [file genes-13-00150-s001.zip › Figure S2.pdf]

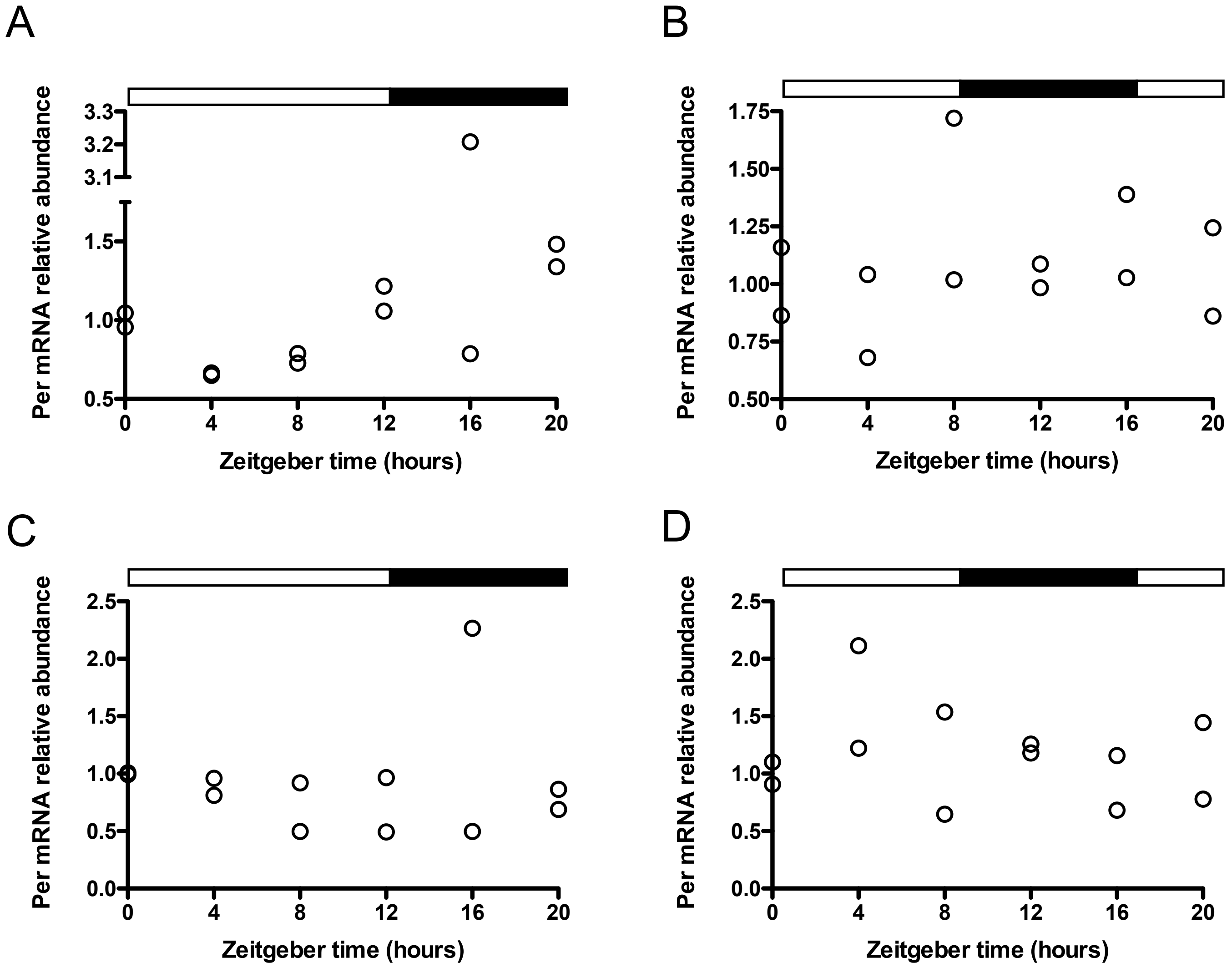

Figure S2. RT-qPCR expression analysis of *period* gene expression. Bars represent light and dark periods. (a) Relative expression of the *period* gene under 12:12 L:D conditions. Each data point represents one independent sample carried out with three technical replicates. The expected gene expression cycling is seen, but with a four hour phase delay compared to the expected phase. (b) Relative expression of the *period* gene under 8:8 L:D conditions. Each data point represents one independent sample carried out with three technical replicates. No clear rhythmicity is seen and substantial inter-sample variability is apparent. (c) No RT (reverse transcriptase) control of 12:12 L:D samples. Amplification suggests some contamination with genomic DNA, but cycling is not evident. The outlier is not the same sample as in panel (a). Each data point represents one technical replicate of an independent sample with a four-fold excess of template compared to the experimental reactions. (d) No RT control of 8:8 L:D samples. Amplification suggests some contamination with genomic DNA, but the pattern does not match panel b, suggesting that results in panel b reflect differences in gene expression, not genomic DNA contamination. Each data point represents one technical replicate of an independent sample with a four-fold excess of template compared to the experimental reactions. The data file used to generate these figures is available as Table S3.
